# Fabrication of Zein Nanoparticle-Functionalized Wheat Gluten Amyloid Fibril/Methyl Cellulose Hybrid Membranes with Efficient Performance for Water-in-Oil Emulsion Separation

**DOI:** 10.3390/polym17172409

**Published:** 2025-09-04

**Authors:** You-Ren Lai, Jun-Ying Lin, Jou-Ting Hsu, Ta-Hsien Lin, Su-Chun How, Steven S.-S. Wang

**Affiliations:** 1 Department of Chemical Engineering, National Taiwan University, Taipei 106319, Taiwan; ray110135@gmail.com (Y.-R.L.); kuroha56780@gmail.com (J.-Y.L.); 710416wlsh.tyc.edu.tw@gmail.com (J.-T.H.); 2 Institute of Biochemistry and Molecular Biology, National Yang Ming Chiao Tung University, Taipei 11221, Taiwan; thlin@vghtpe.gov.tw; 3 Department of Chemical Engineering and Biotechnology, Tatung University, Taipei 10452, Taiwan

**Keywords:** amyloid fibril, nanoparticle, three-dimensional membrane, emulsion separation, wheat gluten, zein

## Abstract

Considering the high stability of water-in-oil (W/O) emulsions, contamination from emulsified pollutants poses a long-term risk to the environment. In this study, hybrid membranes composed of wheat gluten amyloid fibrils (WGAFs) and zein nanoparticles (ZNPs) were prepared and used as a separator to remove emulsified W/O droplets from the oily phase. ZNPs and WGAFs were synthesized through antisolvent method and fibrillation process. Next, a ZNP-functionalized wheat gluten AF/methyl cellulose (ZNP-WGAF/MC) hybrid membrane was fabricated, and its properties were investigated via various analytical techniques. Lastly, the separation efficiency of the ZNP-WGAF/MC hybrid membrane for various W/O emulsions was assessed using microscopy and light scattering. The formation of ZNPs or WGAFs was first verified via spectroscopic and microscopic methods. Our results indicated that the ZNP-WGAF/MC hybrid membranes were synthesized via chemical crosslinking coupled with the casting method. Furthermore, the incorporation of either WGAFs or ZNPs was found to improve the thermal stability and surface hydrophobicity of membranes. Finally, the separation efficiency of the ZNP-WGAF/MC hybrid membranes for various W/O emulsions was determined to be ~87–99%. This research demonstrates the potential of harnessing three-dimensional membranes composed of plant protein-based fibrils and nanoparticles to separate emulsified W/O mixtures.

## 1. Introduction

In an era of rapid industrialization and urbanization, environmental issues resulting from the discharge of industrial oily wastewater have harmful effects on the ecosystem and pose serious risks to human health [1,2,3]. Thus, the separation of emulsified oil/water mixtures is a critical process in various industries, including metal and food processing, wastewater treatment, pharmaceutical manufacturing, and the oil and gas industry [1,2,3]. Once oily wastes are discharged into the aquatic environment, the oil/water mixture, in the form of either an immiscible or emulsified mixture, persists in the environment and leads to long-term contamination [1,4]. The separation of immiscible oil/water mixtures can be efficiently achieved through gravity settling based on the density difference between the two substances. In contrast, emulsified oil/water mixtures pose a greater challenge for separation because of their interfacial stability and µm/nm-scale emulsified droplets (generally less than 20 µm) [1,2,3,5]. Given its cost-effectiveness, operational simplicity, and high efficiency, membrane separation has emerged as a promising technology for use in treating emulsion wastewater [6]. Therefore, growing research has explored the synthesis of advanced membranes with hydrophobic/oleophilic properties to effectively remove emulsified droplets from the immiscible continuous phase [1,2,3,5,6].

Their overall high-sustainability footprints, low greenhouse gas (GHG) emissions, minimal environmental impacts, extensive global production, and good renewability and biodegradability make plant proteins promising building blocks for creating green and sustainable membranes [7,8,9]. Wheat gluten (WG) is composed of α-gliadin, γ-gliadin, ω1,2-gliadin, ω5-gliadin, low-molecular-weight glutenin subunits, and high-molecular-weight glutenin subunits [10]. Amyloid fibrils (AFs) belong to a type of protein aggregate characterized by ordered fibrillar structures that are rich in a cross β-sheet secondary structure, and they can be formed via amyloidogenesis of wheat gluten (WG) in recent studies [10,11]. Solid evidence demonstrates that converting protein monomers into AFs has emerged as a promising approach to improving the properties of protein-derived materials, such as increased surface hydrophobicity, enhanced film-forming properties, reinforced mechanical stiffness, and better thermal and chemical stability [4,12]. Zein is rich in alcohol-soluble prolamine and has a high proportion of hydrophobic amino acids. Furthermore, its inherent hydrophobicity, self-assembly tendency, and biocompatibility foster the idea of harnessing zein as an ideal candidate for nanoparticle preparation [13,14]. Recent research has reported that zein nanoparticles (ZNPs) have been widely utilized in food technology, nanotechnology, and biomedical engineering [4,12,13,14].

In this study, we proposed a three-dimensional membrane system of oleophilic/hydrophobic membranes composed of wheat gluten AFs (WGAFs) and zein nanoparticles (ZNPs) as emulsion separators of oil/water mixtures. WGAFs and ZNPs were synthesized and incorporated into a methyl cellulose (MC) membrane to produce the WGAF/MC hybrid membrane and ZNP-WGAF/MC hybrid membrane for emulsion separation. The size and shape of the ZNPs were analyzed using dynamic light scattering (DLS) and transmission electron microscopy (TEM). The morphology and secondary structure composition of the WGAFs were examined via TEM and Fourier-transform infrared spectroscopy (FTIR). We performed FTIR, scanning electron microscopy (SEM), and thermogravimetric analysis (TGA) to investigate the functional groups, surface microstructure, and thermal properties of the ZNP-WGAF/MC hybrid membrane. The separation performances of the hybrid membranes toward varying W/O emulsions were evaluated using optical microscopy (OM), DLS, and right-angle light scattering (RA). Furthermore, the influence of membrane composition on the emulsion separation efficiency of the hybrid membrane was examined accordingly. This study demonstrates the possibility of nanoparticle-amyloid fibril membrane systems for water-in-oil emulsion separation.

## 2. Materials and Methods

### 2.1. Materials

Zein protein, wheat gluten (WG), methyl cellulose (MC), glutaraldehyde (GA), and thioflavin-T (ThT) were purchased from Sigma-Aldrich (St. Louis, MO, USA). Hydrochloric acid (HCl) and potassium bromide (KBr) were obtained from J.T. Baker (Philadelphia, PA, USA). Tween 80 was acquired from Fisher Biotech (Waltham, MA, USA). Gasoline (GOL) and diesel oil (DSO) were purchased from the Chinese Petroleum Corporation (Taipei, Taiwan). Soybean oil (SBO) and sunflower oil (SFO) were obtained from Uni-President Enterprises Corporation (Tainan, Taiwan). Gear oil (GRO) was procured from Sanyang Motor Corporation (Taipei, Taiwan).

### 2.2. Preparation of Zein Nanoparticles (ZNPs)

The zein nanoparticles (ZNPs) were synthesized using the antisolvent method [15,16]. 120 mg of zein protein powder was first dissolved in 5 mL of an 80% ethanol solution to yield 24 mg/mL zein solution. Next, the zein solution was added dropwise to 15 mL of deionized (DI) water at 1 mL/min. Finally, the zein solution and DI water were mixed with a stirring speed of 1200 rpm to form a solution containing 6 mg/mL zein ZNPs.

### 2.3. Synthesis of Wheat Gluten Amyloid Fibrils (WGAFs)

A WG solution (200 mg/mL) was prepared by dissolving 600 mg of WG powder in 3 mL of 70% ethanol. The WG solution was vortexed for 2 min, sonicated for 5 min, vortexed again for 1 min, and centrifuged at 5000 rpm for 6 min. The supernatant, containing gliadin, was removed, and the precipitates were dissolved in 3 mL of 0.5% SDS-PB solution. Next, the aforesaid mixture was subjected to the same steps to obtain precipitates containing SDS-insoluble WG subunits. Finally, the resulting precipitates were dissolved in 10 mL of HCl solution (pH 1.6) and incubated at 80 °C with a stirring speed of 650 rpm for 48 h to produce WGAFs.

### 2.4. Fabrication of ZNP-Functionalized WGAF/MC Hybrid Membrane

A 100 mg of methyl cellulose (MC) powder was dissolved in 5 mL of a 20% ethanol solution to obtain a 20 mg/mL MC solution. Next, this solution was blended with WGAF. MC concentration ratios of 1:0, 1:1, and 1:2 were prepared at 80 °C with a stirring speed of 650 rpm for 6 h. Furthermore, a 6 mg/mL solution of ZNPs was mixed with the solution blended with WGAF, yielding an MC concentration ratio of 1:1. Afterward, 200 μL of glutaraldehyde (GA) solution were added to the blend solutions as a crosslinker at room temperature with a stirring speed of 650 rpm for 2 h. Finally, the blend solutions were cast onto glass plates and air dried for 3 days to fabricate hybrid membranes. The WGAF/MC hybrid membranes with WGAF:MC concentration ratios of 1:0, 1:1, and 1:2 were named the MC membrane, the WGAF/MC-1 membrane, and the WGAF/MC-2 membrane. Furthermore, the ZNP-functionalized WGAF/MC-1 membrane was designated as the ZNP-WGAF/MC membrane.

### 2.5. Thioflavin T (ThT) Fluorescence Assay

10 μM Thioflavin T (ThT) solution was initially prepared following the published experimental procedure [17,18]. The WGAF sample solution was mixed with the ThT solution at a volume ratio of 1:24 and then placed into a 1 cm light path quartz cuvette for fluorescence measurement. The fluorescence spectrophotometer (Cary Eclipse 300, Varian, Palo Alto, CA, USA) was used at an excitation wavelength of 440 nm to measure the ThT fluorescence intensity of WGAF samples at emission wavelengths ranging from 460 to 550 nm. The excitation and emission slit widths and photomultiplier tube (PMT) voltage were set to 5 nm and 600 V.

### 2.6. Fourier-Transform Infrared Spectroscopy (FTIR)

Each sample was first dehydrated by drying at 60 °C for one day. Next, the samples were ground and mixed with potassium bromide at a weight ratio of 1:100, then compressed into tablets. Finally, a Fourier-transform infrared (FTIR) spectrometer (Spectrum GX, Perkin Elmer, Shelton, CT, USA) was employed to obtain the FTIR spectra of the samples in the wavenumber range from 500 to 4000 cm^−1^.

### 2.7. Thermogravimetric Analysis (TGA)

The samples were dried at 60 °C for one day to remove excess moisture. Then, 5 mg of each sample was weighed and placed in a platinum crucible. A thermogravimetric analyzer (PYRIS 1, Perkin Elmer, Shelton, CT, USA) was used to conduct thermogravimetric (TGA) analysis, with the following settings: a heating rate of 10 °C per minute from 50 to 750 °C in a nitrogen atmosphere.

### 2.8. Contact Angle Goniometry

7 μL of deionized (DI) water was placed on the surface of the membrane samples. After adjusting the aperture and focus, the membrane samples’ water contact angles (θ) were measured using a contact angle goniometer (FTA125, First Ten Ångstroms, Portsmouth, VA, USA). Finally, the water contact angles (θ) of the membrane samples before and after emulsion separation were measured and compared.

### 2.9. Dynamic Light Scattering (DLS)

1 mL of sample solution was placed in a disposable polystyrene cuvette with a 10 mm light path. The dynamic light scattering (DLS) analysis of samples was conducted using a Zetasizer instrument (Zetasizer Pro, Malvern Panalytical, Westborough, MA, USA) at a laser wavelength of 633 nm and a fixed scattering angle of 173°. The hydrodynamic size distribution, average hydrodynamic diameter (D_h_), and polydispersity index (PDI) of the samples were determined through triplicate measurements.

### 2.10. Transmission Electron Microscopy (TEM)

10 μL of WGAF solution was pipetted onto the surface of carbon-stabilized copper grids and left to stand for 10 s. The copper grids placed with WGAF were negatively stained with 1% (*w*/*v*) uranyl acetate (UA) for 90 s. After air-drying for 20 min, the WGAF placed on the copper grid was investigated and photographed using a transmission electron microscopy (TEM) equipped with a Gantan model 782 CCD camera (H-7650, Hitachi, Tokyo, Japan) at an accelerating voltage of 75 kV.

### 2.11. Scanning Electron Microscopy (SEM)

The membrane samples were air-dried for a day and then coated with platinum using the magnetron sputtering system (MSP-1S, Vacuum Device Inc., Mito, Japan). Scanning electron microscopy (FlexSEM 1000 II, Hitachi, Tokyo, Japan) was used to examine and photograph the platinum-coated membranes’ surface and internal microstructures.

### 2.12. Water-in-Oil Emulsion Separation Test

The water-in-oil (W/O) emulsions were first synthesized by mixing DI water with various oils at a volume ratio of 1:99. Next, Tween 80 was added to the water/oil mixture at a surfactant: total volume ratio of 1:500 and then homogenized through ultrasonication to produce emulsified W/O droplets. After that, the W/O emulsions were passed through hybrid membranes, and the resulting filtrate was analyzed to determine particle size using DLS and optical microscope (OM) (ECLIPSE E200, Nikon, Tokyo, Japan). Finally, the right-angle light scattering intensity at 450 nm of W/O emulsions and the resulting filtrate were measured using a fluorescence spectrophotometer (Cary Eclipse 300, Varian, Palo Alto, CA, USA), and the separation efficiency of the hybrid membranes was evaluated using the following equation:

(1) $$\mathrm{Separation}\;\mathrm{efficiency}\;(\mathrm{SE}\%)\;=\;\left(\frac{{\mathrm{RA}}_{i}-{\mathrm{RA}}_{f}}{{\mathrm{RA}}_{i}}\right)\;\times \;100\;\%$$

Here, RA_i_ and RA_f_ denote the right-angle light scattering intensities of the W/O emulsion sample before and after filtration, respectively.

### 2.13. Statistical Analysis

All experimental data obtained from n independent determinants were calculated and presented as the mean ± standard deviation (SD). Unless otherwise specified, statistically significant differences in the experimental results were determined using Student’s *t*-test with a two-tailed analysis and an independent two-sample *t*-test. Statistical significance was considered as *p* < 0.05, indicating a significant difference. The statistical analyses were performed using Origin2024 (OriginLab, Northampton, MA, USA) and Microsoft 365 excel software (Microsoft, Redmond, WA, USA).

## 3. Results and Discussion

### 3.1. Formation and Characterization of Zein Nanoparticles (ZNPs)

**Figure 1:**
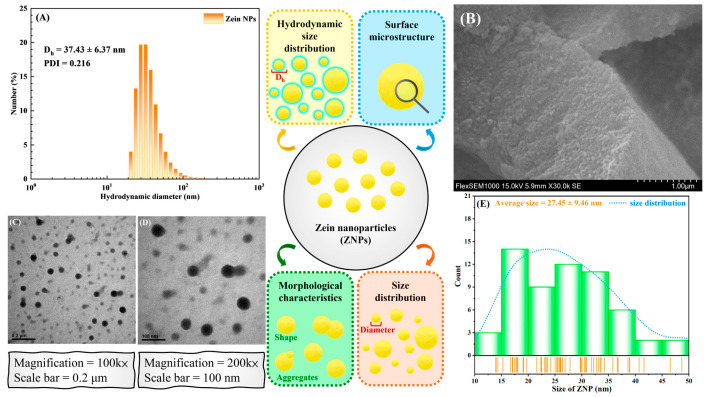
(**A**) Hydrodynamic size distribution of ZNPs. (**B**) SEM micrograph of ZNPs. (Scale bar = 1 μm) TEM micrograph of ZNPs at magnifications of (**C**) 100 k× and (**D**) 200 k×. (**E**) Diameter distribution of ZNPs as analyzed by ImageJ.

To verify the formation of ZNPs, we performed DLS, TEM, and SEM to investigate their size distribution, morphology, and surface microstructure, respectively. As shown in Figure 1A, the ZNPs exhibited a narrow hydrodynamic size distribution. The average hydrodynamic diameter (D_h_) and polydispersity index (PDI) of the ZNPs were determined to be 37.43 ± 6.37 nm and 0.216, respectively. The PDI is employed as a measure of the heterogeneity and broadness of particle size distribution. The low PDI value of ZNPs indicated unimodal and monodisperse particle size distribution, reflecting that the ZNPs have a homogeneous size distribution, uniform dispersity, and good colloidal stability in solution [19,20]. Observations via SEM revealed that large aggregates were composed of numerous ZNPs in the form of solid powder particles (see Figure 1B). Figure 1C,D depict the morphology of the ZNPs, specifically noting that they possess a spherical shape and a nm-scale diameter. Based on the analysis of the TEM micrograph results obtained using ImageJ software (version 1.54d, Wayne Rasband, National Institute of Health, Bethesda, MD, USA), the size distribution and average diameter of the ZNPs were determined, which were consistent with our DLS findings (see Figure 1E). Our results indicate that ZNPs were successfully produced.

### 3.2. Synthesis and Characterization of Wheat Gluten Amyloid Fibrils (WGAFs)

**Figure 2:**
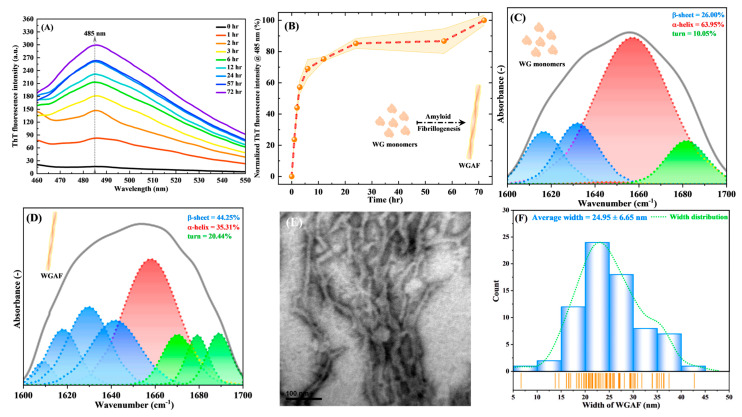
(**A**) ThT fluorescence spectra of WGAFs at different incubation times (excitation wavelength = 440 nm). (**B**) ThT fluorescence intensity at an emission wavelength of 485 nm of WGAFs as a function of incubation times. FTIR deconvolution spectra of (**C**) wheat gluten monomers and (**D**) WGAFs. (**E**) TEM micrograph of WGAFs (Scale bar = 100 nm). (**F**) Width distribution of WGAFs as analyzed by ImageJ.

The high-molecular-weight glutenin subunit (HMW-GS) in wheat gluten has been shown to have a propensity for amyloid fibrillation [21]. The SDS-PAGE results in Figure S1 demonstrate the successful purification of HMW-GS from wheat gluten through a two-stage purification process. To verify the synthesis of bona fide WGAFs, the ThT binding assay was used to detect WGAFs. Since ThT molecules, a fluorescence probe, emit strong fluorescence intensity at ~485 nm upon specifically binding to cross β-sheet structures of amyloid fibrils, the ThT fluorescence assay is extensively used to verify amyloid fibrillogenesis of proteins [17,18]. Figure 2A,B show a marked increase in the ThT fluorescence intensity of WG with incubation time, indicating the formation of mature WGAFs. The FTIR deconvolution spectra of WG monomers and WGAFs are shown in Figure 2C,D. The amide I region, at 1600–1700 cm^−1^, which appeared in the FTIR spectra of proteins, corresponds to the vibration of carbonyl group (C=O) stretching and can help determine the secondary structure composition of proteins [17]. Moreover, various peaks at 1600–1635 cm^−1^, 1645–1665 cm^−1^, and 1666–1700 cm^−1^ that appeared in the amide I region correspond to β-sheet, α-helix, and turn, respectively [15,22]. Our results indicate the secondary structure of the WG monomers (∼64% α-helix and ∼26% β-sheet) and WGAFs (∼35% α-helix and ∼44% β-sheet), suggesting that WG amyloid fibrillogenesis is associated with an increase in β-sheet secondary structure. As depicted in Figure 2E,F, the morphology of WGAFs exhibited a filamentous structure with a high length-to-width ratio. Additionally, the average width of the WGAFs was found to be 24.95 ± 6.65 nm, which is consistent with the structural features of WGAFs reported in previous studies [10]. These results indicated that mature WGAFs were successfully synthesized.

### 3.3. Surface Chemistry and Thermal Properties of ZNP-WGAF/MC Hybrid Membranes

**Figure 3:**
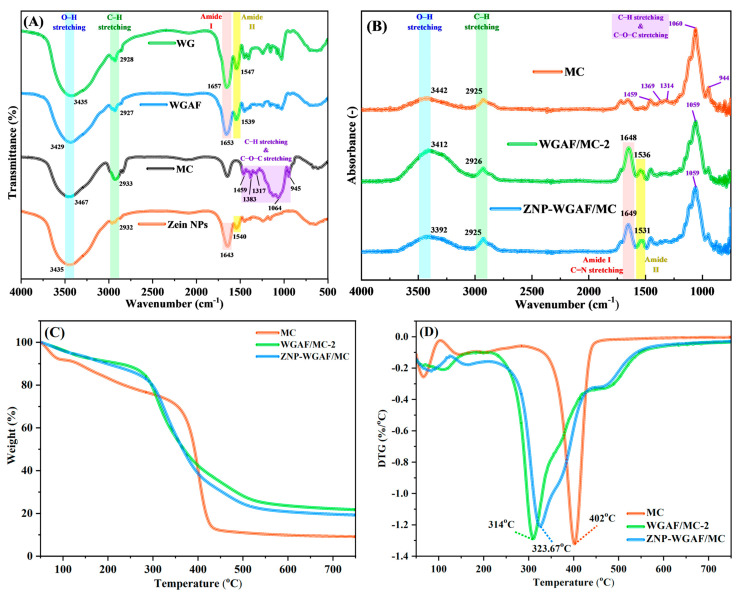
(**A**) FTIR spectra of wheat gluten monomers, MC, WGAFs, and ZNPs. (**B**) ATR-FTIR spectra of MC membrane, WGAF/MC hybrid membrane, and ZNP-WGAF/MC hybrid membrane. (**C**) TGA and (**D**) DTG curves of MC membrane, WGAF/MC hybrid membrane, and ZNP-WGAF/MC hybrid membrane.

The FTIR spectra of WG monomers, WGAFs, MC, and ZNPs are shown in Figure 3A. The characteristic peaks at ~1657, ~1653, and ~1643 cm^−1^ in the FTIR spectra of WG monomers, WGAFs, and ZNPs corresponded to the vibration of C=O stretching [4,17,23]. Peaks appeared at ~1547, ~1539, and ~1540 cm^−1^ in the FTIR spectra of WG monomers, WGAFs, and ZNPs, indicating the amide II of polypeptide backbones [4]. The peaks at ~3435, ~3429, ~3467, and ~3435 cm^−1^, corresponding to the vibration of O–H group stretching, emerged in the FTIR spectra of WG monomers, WGAFs, MC, and ZNPs. Peaks that represent C−H stretching were observed at ~2928, ~2827, ~2833, and ~2832 cm^−1^ in the FTIR spectra of WG monomers, WGAFs, MC, and ZNPs. For the functional groups of MC, peaks at ~1459, ~1383, ~1317, ~1064, and ~945 cm^−1^ involved the C−H stretching of −CH_2_− and −CH_3_− groups and C−O−C stretching. Figure 3B reveals the ATR-FTIR spectra of the MC membrane, WGAF/MC membrane, and ZNP-WGAF/MC membrane. For the MC membrane, as compared with the WGAF/MC membrane or the ZNP-WGAF/MC membrane, the peaks corresponding to the stretching vibration of O–H groups and C−H groups shifted, indicating the interaction between WGAFs, ZNPs, and MC through hydrogen bonding [24]. Furthermore, the amide II band observed at 1539 cm^−1^ in the FT-IR spectrum of the WGAFs shifted to ~1536 and ~1531 cm^−1^ in the WGAF/MC-2 membrane and the ZNP-WGAF/MC membrane, respectively, indicating that the imine group was formed through the reaction of the WGAF’s N−H groups with the GA’s COOH groups. In the FTIR spectra of the WGAF/MC-2 membrane and ZNP-WGAF/MC membrane, a region containing the characteristic peaks emerged at ~1700–1585 cm^−1^, which involved the C=N stretching of the chemical crosslinking between ZNPs or WGAFs and GA [25]. The TGA and DTG curves of the MC membrane, WGAF/MC membrane, and ZNP-WGAF/MC membrane are shown in Figure 3C,D. When the temperature was increased from 50 to 300 °C, the weight loss of the MC membrane, WGAF/MC membrane, and ZNP-WGAF/MC membrane is due to solvent evaporation [26]. When the temperature increased from 300 to 450 °C, thermal degradation of the MC membrane, WGAF/MC membrane, and ZNP-WGAF/MC membrane was found at ~402, ~314, and ~323 °C, respectively. The better thermal stability of the ZNP-WGAF/MC membrane as compared to the WGAF/MC membrane was attributed to the introduction of ZNPs in the membrane matrix and chemical crosslinking of ZNPs with WGAFs and MC. Furthermore, the weight loss for the WGAF/MC membrane and that for the ZNP-WGAF/MC membrane were lower than that for the MC membrane at 750 °C, indicating that the incorporation of WGAFs or ZNPs would improve the thermal resistance and stability of the membrane [27].

### 3.4. Surface Microstructure and Surface Wettability of ZNP-WGAF/MC Hybrid Membranes

**Figure 4:**
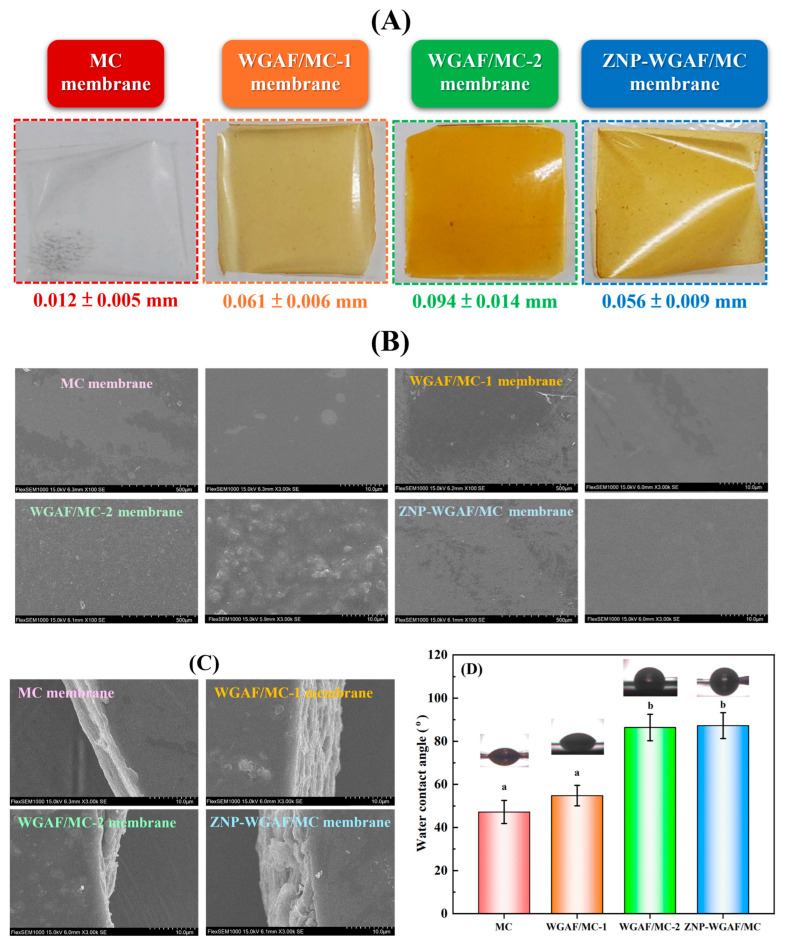
(**A**) Physical appearances and thickness of MC membrane, WGAF/MC hybrid membrane, and ZNP-WGAF/MC hybrid membrane. (**B**) Top-view SEM micrographs of MC membrane, WGAF/MC hybrid membrane, and ZNP-WGAF/MC hybrid membrane. SEM images were taken at magnifications of 100× (left panel) and 3000× (right panel). (**C**) Side-view SEM micrographs of MC membrane, WGAF/MC hybrid membrane, and ZNP-WGAF/MC hybrid membrane. SEM images were taken at magnifications of 3000×. (**D**) Water contact angles of MC membrane, WGAF/MC hybrid membrane, and ZNP-WGAF/MC hybrid membrane. (Different lowercase letters above bars show significant differences (*p* < 0.05)).

Figure 4A illustrates the physical appearance of the MC membrane, the WGAF/MC hybrid membrane, and the ZNP-WGAF/MC hybrid membrane. In contrast to the MC membrane, both the WGAF/MC hybrid membrane and the ZNP-WGAF/MC hybrid membrane exhibited opacity and a yellow color. Furthermore, the color of the WGAF/MC-2 hybrid membrane was deeper than that of the WGAF/MC-1 hybrid membrane, which is attributed to the higher WGAF content in the former. With increased WGAF content, the thickness of the WGAF/MC membrane was increased, which is attributed to a large amount of WGAF being incorporated in the membrane matrix [23,28]. Furthermore, no differences in the thickness of the WGAF/MC-1 hybrid membrane and the ZNP-WGAF/MC hybrid membrane were found, indicating that the addition of ZNPs in the membrane composition may not affect membrane thickness. As depicted in Figure 4B, the surface microstructures of the MC membrane, the WGAF/MC hybrid membrane, and the ZNP-WGAF/MC hybrid membrane are uniform and smooth at a low magnification. Compared to the MC membrane, the WGAF/MC-1 membrane, and the ZNP-WGAF/MC membrane, the WGAF/MC-2 membrane showed a rough surface at a high magnification, which could be attributed to the large amount of WGAF stacked on the membrane surface [23,28]. Moreover, the entanglement of the WGAFs resulted in the formation of an interconnect network of voids on the membrane surface [28]. The internal microstructures of the MC membrane and WGAF/MC hybrid membranes shown in Figure 4C exhibited a hierarchical structure. Upon ZNP incorporation, the ZNP-WGAF/MC hybrid membrane was observed to have a layered internal structure composed of a thick and dense inner layer. Figure 4D reveals the water contact angles (WCAs) of the MC membrane, the WGAF/MC hybrid membrane, and the ZNP-WGAF/MC hybrid membrane. Our results indicated that the WCAs of the MC membrane, WGAF/MC-1 membrane, WGAF/MC-2 membrane, and ZNP-WGAF/MC membrane were ~47°, ~55°, ~86°, and ~87°, respectively, indicating that the WGAF/MC-2 membrane and ZNP-WGAF/MC membrane exhibited better surface hydrophobic properties than the other membranes. This is likely due to the hydrophobic nature of amyloid fibrils and zein protein, which results in the enhanced surface hydrophobicity of the membrane [23,29,30].

### 3.5. Separation Performances of ZNP-WGAF/MC Hybrid Membranes Toward W/O Emulsions

**Figure 5:**
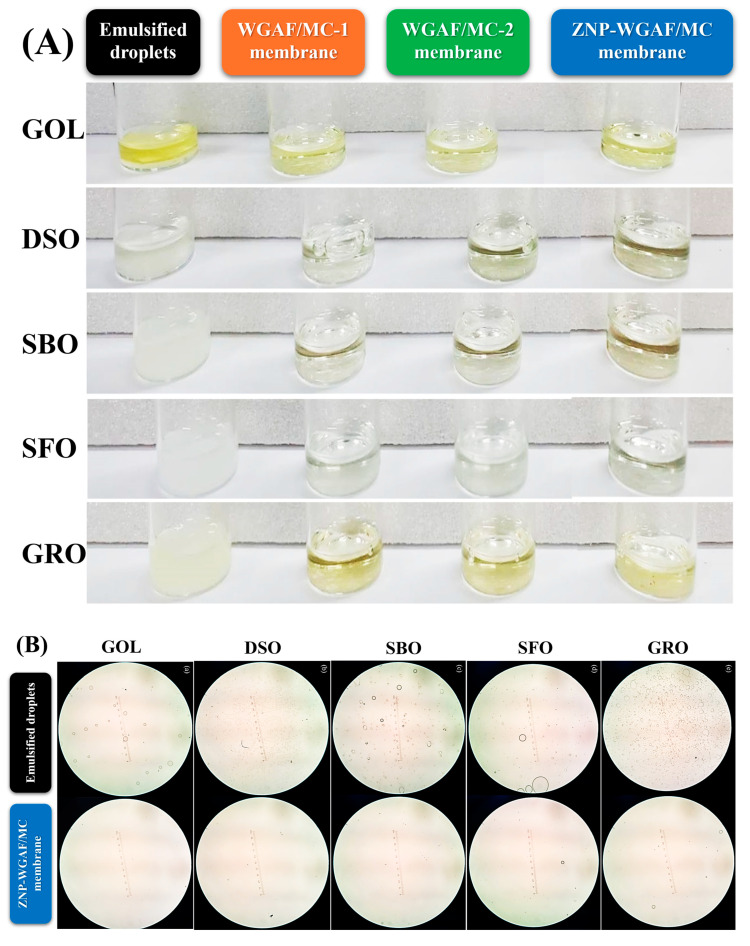

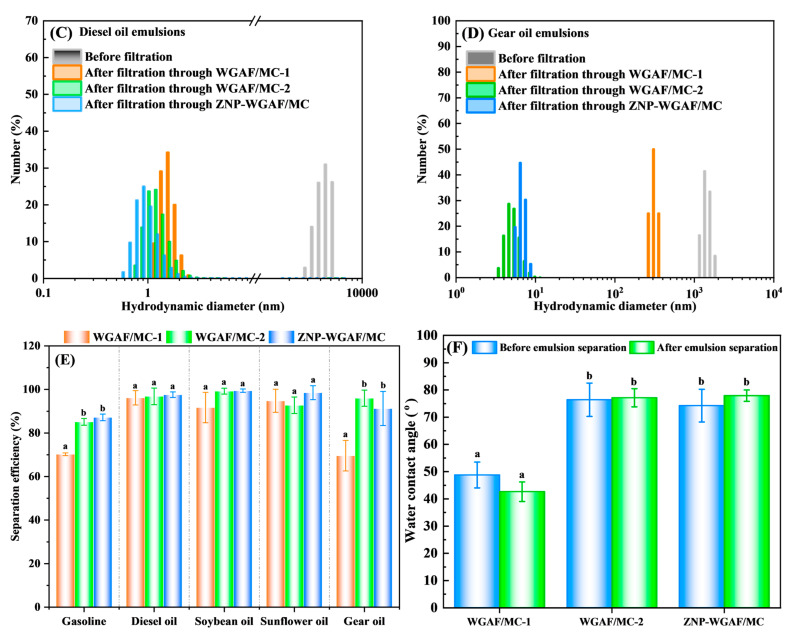
(**A**) Physical appearances and (**B**) optical micrographs of various W/O emulsions before and after filtration through WGAF/MC hybrid membrane and ZNP-WGAF/MC hybrid membrane. (GOL: gasoline, DSO: diesel oil, SBO: soybean oil, SFO: sunflower oil, and GRO: gear oil) Hydrodynamic size distributions of the (**C**) W/DSO emulsion and (**D**) W/GRO emulsion before and after filtration through WGAF/MC hybrid membrane and ZNP-WGAF/MC hybrid membrane. (**E**) Separation performances of the WGAF/MC hybrid membrane and ZNP-WGAF/MC hybrid membrane for various emulsified W/O droplets. (**F**) Water contact angles of the WGAF/MC hybrid membrane and ZNP-WGAF/MC hybrid membrane before and after filtering emulsified W/DSO mixtures. (Different lowercase letters above bars show significant differences (*p* < 0.05)).

Given their high stability and the difficulty of removing them from water, emulsified W/O droplets can significantly contaminate aquatic environments and ecosystems [31]. Thus, the ZNP-WGAF/MC hybrid membranes developed in this study were utilized to eliminate emulsified W/O droplets, evaluating their separation efficiencies against W/O emulsions. As depicted in Figure 5A,B, the physical appearances and optical micrographs of W/O emulsions prepared using various oils were observed. Before filtration through hybrid membranes, the physical appearances of all W/O emulsion solutions were observed to have high turbidity. Moreover, a large amount of μm-sized emulsified W/O droplets were observed in optical micrographs. In contrast, the physical appearance of all W/O emulsion solutions after filtration through hybrid membranes reveals high transparency, while no or fewer emulsified W/O droplets were found using optical microscopy. It is evident that W/O emulsions can be effectively removed from the oil phase via filtration through the ZNP-WGAF/MC hybrid membrane.

Figure 5C,D reveal the hydrodynamic diameter distributions of the W/O emulsions prepared using DSO or GRO before and after filtration using hybrid membranes. Our results show that the droplet diameters of the W/DSO emulsions and the W/GRO emulsions were in the range of ~2000–6000 nm and ~1000–2000 nm, respectively. Upon filtration through the WGAF/MC-1 membrane, the droplet diameters of the W/DSO emulsions and the W/GRO emulsions had decreased to ~200–400 nm. Compared to the WGAF/MC-1 membrane, the droplet diameters of the W/O emulsions filtered through the WGAF/MC-2 membrane and ZNP-WGAF/MC hybrid membrane were lower, which can be attributed to the greater surface hydrophobicity of the WGAF/MC-2 membrane and ZNP-WGAF/MC hybrid membrane. Furthermore, the droplet diameters of W/DSO emulsions and the W/GRO emulsions after filtration through the WGAF/MC-2 membrane or ZNP-WGAF/MC membrane had decreased to the range of ~1–10 nm, indicating that the larger sizes of W/O emulsions were removed through the demulsification and size-sieving effect [31,32]. As a control, the same experimental procedure for separation of various W/O emulsions using the MC membrane was performed to assess the emulsion separation performance of the MC membrane. As depicted in Figure S2 and Table S1, the average hydrodynamic diameters of various W/O emulsions filtered through WGAF/MC and ZNP-WGAF/MC hybrid membranes were markedly smaller than those filtered through the MC membrane, indicating that incorporation of either WGAFs or ZNPs into the membrane matrix can effectively separate large-size W/O emulsions from the oil phase. Furthermore, the average hydrodynamic diameters of various W/O emulsions filtered through the WGAF/MC-2 membrane and ZNP-WGAF/MC hybrid membrane were smaller than those of the WGAF/MC-1 membrane, likely due to higher surface hydrophobicity and thickness of membranes.

Figure 5E shows the separation efficiencies of the WGAF/MC membrane and ZNP-WGAF/MC hybrid membrane for various emulsified W/O droplets, revealing no significant difference in separation efficiency among different hybrid membranes for W/SFO, W/DSO, and W/SBO emulsions. For W/GOL and W/GRO emulsions, the separation efficiencies of the WGAF/MC-2 membrane and the ZNP-WGAF/MC hybrid membrane exceeded 85%. In contrast, the WGAF/MC-1 membrane showed lower separation efficiencies. The high WGAF content of the membrane and incorporation of ZNPs in the membrane structure likely enhanced the membrane’s surface hydrophobicity, contributing to the higher separation efficiencies of the WGAF/MC-2 and ZNP-WGAF/MC membranes compared to the WGAF/MC-1. Our right-angle light scattering results show that incorporating WGAFs or ZNPs can increase surface hydrophobicity and improve emulsion separation performance of hybrid membranes, making them effective at removing emulsified W/O droplets from the oil phase. Presented in Figure 5F are the WCAs of both the WGAF/MC hybrid membrane and the ZNP-WGAF/MC hybrid membrane before and after filtering emulsions. It evidently shows that there are no significant effects on the surface wettability of these membranes, suggesting the potential reusability of both the WGAF/MC and ZNP-WGAF/MC hybrid membranes [23]. Compared to WGAF/MC and ZNP-WGAF/MC hybrid membranes, the separation performance of the MC membrane against various emulsions was relatively lower (see Figure S3). Additionally, a decrease in the WCA of the MC membrane after emulsion separation was observed, indicating that the MC membrane is not suitable for reuse in emulsion separation due to membrane fouling [33]. Taken together, the incorporation of WGAFs and ZNPs is indispensable for better separation performance and reusability of the membrane.

**Figure 6:**
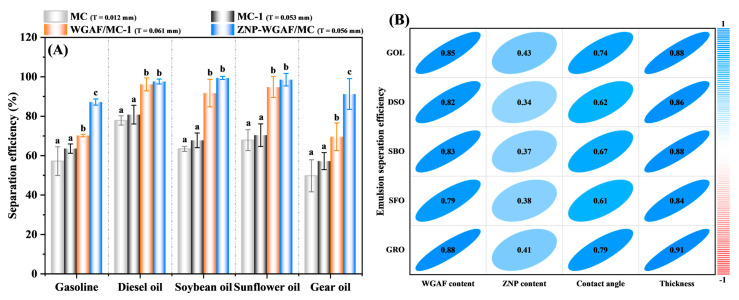
(**A**) Separation performances of the MC, MC-1, WGAF/MC-1, and ZNP-WGAF/MC membranes for various emulsified W/O droplets (The capital letter T denotes membrane thickness) (Different lowercase letters above bars show significant differences (*p* < 0.05)). (**B**) Correlation between the membrane composition and properties with emulsion separation efficiency, as determined using Pearson correlation coefficients.

To examine whether the membrane thickness primarily affects the emulsion separation efficiency of the MC membrane, we prepared the MC-1 membrane with a thickness (T) similar to that of WGAF/MC-1 and ZNP-WGAF/MC membranes. As shown in Figure 6A, the average separation efficiencies of the MC-1 membrane toward various W/O emulsions were better than those of the MC membrane. However, there are no statistical differences in emulsion separation efficiency between MC and MC-1 membranes, signifying that the emulsion separation efficiency of the MC membrane was not enhanced with membrane thickness. The inverse results regarding the effect of membrane thickness on emulsion separation were observed between the MC and WGAF/MC membranes, indicating that the emulsion separation efficiency of membranes depended on the membrane’s materials, thickness, and surface wettability [34,35]. Thus, the emulsion separation efficiency of the MC-1 membrane was still lower than that of the WGAF/MC-1 and ZNP-WGAF/MC membranes, even though the membranes had similar thickness. We show in Figure 6B that the correlation between membrane composition and properties with emulsion separation efficiency demonstrates a positive relationship (Pearson correlation coefficients > 0). This indicated that factors such as WGAF content, ZNP content, contact angle, and thickness influence the emulsion separation efficiency of membranes for each respective oil type. As a result, we suspect that the increase in WGAF content, ZNP content, contact angle, and thickness could potentially enhance the membranes’ emulsion separation performance.

### 3.6. Discussion

For comparison purposes, the average diameter of the zein NPs reported in this study and other previously published zein NPs is listed and summarized in Table S2. Various synthesis methods have been used to produce zein NPs, such as the antisolvent, pH-cycle, pH-driven, and microfluidic approaches [16,36,37,38,39,40,41]. Antisolvent precipitation is the most common method used to produce a wide range of hydrodynamic particle sizes, from ~37.43 nm in this study to ~200 nm in zein nanoparticles encapsulating bioactive compounds. Previous research has demonstrated that zein nanoparticles complexed with sodium caseinate (NaCas) were synthesized via the pH-cycle method, resulting in particles with a hydrodynamic diameter of ~96.9 nm. Furthermore, zein nanoparticles stabilized by whey protein nanofibrils were produced through a pH-driven method, exhibiting a hydrodynamic size of ~410 nm. A study found that a microfluidic approach synthesized PEGylated zein nanoparticles with a hydrodynamic diameter of ~133.3 nm. As a result, the hydrodynamic diameter (D_h_) of ZNPs reported in this study was smaller than those of other published ZNPs.

Recent studies on the development of functional materials for emulsion separation have gained notable attention [4,42,43,44,45,46,47,48]. Furthermore, increasing research demonstrates that various functional materials (e.g., membranes, aerogels, particles, and sponges) have been synthesized and used to remove emulsified droplets from immiscible phases. A summary of emulsion separation systems using different materials is shown in Table S3. A detailed description regarding various emulsion separation systems is given below: (a) The T-SA/lignin^x^/rGO-MTMS aerogel membrane synthesized via chelation and chemical vapor deposition has been employed for chloroform-water separation, and its separation efficiency was found to be ~96.7% [42]. (b) The C_18_-CQDs membrane prepared using thermal decomposition has been demonstrated to possess high separation efficiency (>99%) for both hexane-water and dodecane-water mixtures [43]. (c) Evidence has indicated that cotton, beeswax, and lignin have been utilized to form biomass-based porous materials and shown to be effective in trichloromethane-water separation, with a separation efficiency of ~93.78% [44]. (d) The TDA-MXene@melamine sponge prepared using surface functionalization and vacuum drying has been used to separate water-in-toluene and water-in-dichloromethane mixtures, reaching high separation efficiencies of ~96.88% and ~97.10% [45]. (e) The hybrid material composed of SiO_2_ and DVB was fabricated via the solvothermal method. After filtration through hybrid materials, the droplet sizes of water-in-toluene, water-in-chloroform, and water-in-hexane emulsions were decreased to ~1 nm, 40–100 nm, and 100–1000 nm [46]. (f) A study demonstrated that green fluoride-free superhydrophobic hierarchical flowerlike iron-containing MnO_2_ particles used for diesel-in-water and isooctane-in-water separation were synthesized using a one-pot approach coupled with stearic acid modification [47]. (g) Evidence has shown that filtration devices loaded with treated bamboo powder have been used to remove various water-in-oil emulsions. It demonstrated that the emulsion separation system exhibited high separation efficiency (>99%), and small emulsified droplet sizes (~0.5–40 nm) were determined after treatment with filtration devices [48]. (h) The polysaccharide-modified whey protein amyloid fibril (WPIAF) aerogels used in emulsion separation were prepared through a salting-out method combined with lyophilization. Moreover, the polysaccharide-modified WPIAF aerogels demonstrated high separation efficiency (>90%) across various emulsion systems, and the emulsion sizes were reduced to <20 nm after filtration through the aerogels [4].

Despite the fact that conventional polymeric materials have been extensively employed in oil/water separation, these materials still present several problems/concerns, such as a lack of biocompatibility and biodegradability; production of secondary pollutants, microplastics, and hazardous products; and non-renewability [49,50,51,52]. The above-mentioned shortcomings arising from polymeric materials could considerably restrict their application in oil/water separation. To address the aforesaid limitation, significant efforts have been made to develop sustainable functional materials as oil/water separators using bio-based, renewable, and biodegradable components (e.g., lignin, alginate, cotton, beeswax, lignin, bamboo, whey protein, cellulose, chitosan) [4,42,44,48]. In our study, three-dimensional membranes composed of byproduct wheat gluten and zein from the milling processes of rice and maize starch were successfully synthesized, and the possibility of harnessing these membranes as emulsion separators was examined accordingly. In terms of protein waste valorization, our study demonstrates that wheat gluten and zein can be converted into amyloid fibrils and nanoparticles, which can serve as building blocks for functional membranes [7]. Among all materials listed in Table S3, our WGAF/MC and ZNP-WGAF/MC membranes demonstrated comparable or superior performance to other published materials from the perspectives of emulsion separation efficiency and droplet size after filtration, highlighting the applicability of WGAF/MC and ZNP-WGAF/MC membranes in the emulsion separation. Despite the advantageous properties of the ZNP-WGAF/MC membrane system developed in this study, it is important to acknowledge certain drawbacks/limitations of this system for the sake of completeness. Our results indicated that the WGAF/MC and ZNP-WGAF/MC membranes exhibited water contact angles (WCAs) of ~86° and ~87°, respectively, which are higher than that of the bare MC membrane (~47°). However, the surface hydrophobicity of WGAF/MC and ZNP-WGAF/MC membranes is not as good as other materials with superhydrophobicity (WCA > 150°) and superoleophilicity (contact angle between oil and surface ~ 0°) [53]. To address the limitations of WGAF/MC and ZNP-WGAF/MC membranes with low surface hydrophobicity, chemical modification of the membrane surface through grafting low-surface-energy materials could enhance surface hydrophobicity and improve performance in emulsion separation [54]. In our future research, we will focus on the implementation of surface modification in the synthesis of protein-based membranes, aiming to enhance the separation performance of the protein-based membrane system.

## 4. Conclusions

In this study, we aimed to fabricate a three-dimensional ZNP-functionalized WGAF/MC hybrid membrane as a separator and examine its potential for emulsion separation. First, ZNPs and WGAFs were formed through the antisolvent method and the fibrilization process, followed by characterizing their properties using various analytical techniques. Next, the ZNP-functionalized WGAF/MC hybrid membrane was synthesized using chemical crosslinking coupled with the casting method. Moreover, the surface chemistry, microstructure, thermal properties, and wettability of the ZNP-WGAF/MC hybrid membrane were investigated. The effects of WGAF content and ZNP incorporation on the thermal degradation, microstructure, and hydrophobicity of the membrane were assessed. Our emulsion separation results indicate that the ZNP-WGAF/MC hybrid membrane can effectively remove emulsified W/O droplets from the oily phase due to its demulsification and size-sieving effect. Furthermore, the ZNP-WGAF/MC hybrid membrane demonstrated excellent separation efficiency (~85–99%) for various W/O emulsions. Upon filtration through the ZNP-WGAF/MC membrane, the hydrodynamic diameters of the emulsified droplets were reduced from a μm scale to a nm scale. This study shows the development of a nanoparticle-amyloid fibril hybrid membrane as a separator for the removal of emulsion contaminants.

## Data Availability

The original contributions presented in this study are included in the article/Supplementary Material. Further inquiries can be directed to the corresponding authors.

## Supplementary Materials

The following supporting information can be downloaded at: https://www.mdpi.com/article/10.3390/polym17172409/s1, Table S1: Estimate of average hydrodynamic diameters for various emulsions before and after filtering through MC, WGAF/MC-1, WGAF/MC-2, and ZNP-WGAF/MC membranes; Table S2: A summary of the synthesis methods and particle sizes of various zein nanoparticles; Table S3: Comparison of the performances of emulsion separation systems using various materials; Figure S1: (A) SDS-PAGE analyses of WG (Lane 1: unpurified WG (6 mg/mL); Lane 2: supernatant obtained after purification with a 70% ethanol solution (6 mg/mL); Lane 3: supernatant obtained after purification with a 0.5% SDS-PB buffer (6 mg/mL); Lane 4: purified WG (6 mg/mL); Lane 5: supernatant obtained after purification with a 0.5% SDS-PB buffer (24 mg/mL)); Figure S2: Hydrodynamic size distributions of the W/O emulsions (A: diesel oil and B: gear oil) before and after filtration through MC membrane; Figure S3: (A) Separation performances of the MC membrane for various emulsified W/O droplets. (B) Water contact angles of the MC membrane before and after filtering emulsified W/DSO mixtures. (Different lowercase letters above bars show significant differences (*p* < 0.05)).

## References

[1] Zhang N., Yang X., Wang Y., Qi Y., Zhang Y., Luo J., Cui P., Jiang W. (2022). A review on oil/water emulsion separation membrane material. J. Environ. Chem. Eng..

[2] Wu J., Wei W., Li S., Zhong Q., Liu F., Zheng J., Wang J. (2018). The effect of membrane surface charges on demulsification and fouling resistance during emulsion separation. J. Membr. Sci..

[3] Peng Y., Guo Z. (2016). Recent advances in biomimetic thin membranes applied in emulsified oil/water separation. J. Mater. Chem. A.

[4] Tu J.-L., Lai Y.-R., Lin C.-Y., Wang S.S.-S., Lin T.-H. (2024). Applications of three-dimensional whey protein amyloid fibril-based hybrid aerogels in oil/water separation and emulsion separation. Int. J. Biol. Macromol..

[5] Zhang J., Lao X., Jiang X., Li Z., Feng W., Chen L. (2024). Sheep bone powder modified PVDF membrane for highlyefficient oil-in-water emulsion separation. J. Taiwan Inst. Chem. Eng..

[6] Li B., Qi B., Guo Z., Wang D., Jiao T. (2023). Recent developments in the application of membrane separation technology and its challenges in oil-water separation: A review. Chemosphere.

[7] Peydayesh M., Bagnani M., Soon W.L., Mezzenga R. (2022). Turning food protein waste into sustainable technologies. Chem. Rev..

[8] Zhou J., Li T., Peydayesh M., Usuelli M., Lutz-Bueno V., Teng J., Wang L., Mezzenga R. (2022). Oat plant amyloids for sustainable functional materials. Adv. Sci..

[9] Rabiee N., Sharma R., Foorginezhad S., Jouyandeh M., Asadnia M., Rabiee M., Akhavan O., Lima E.C., Formela K., Ashrafizadeh M. (2023). Green and sustainable membranes: A review. Environ. Res..

[10] Zhang H., Lv S., Jin C., Ren F., Wang J. (2023). Wheat gluten amyloid fibrils: Conditions, mechanism, characterization, application, and future perspectives. Int. J. Biol. Macromol..

[11] Liang Y., Zhu X., Liu H., Yang L., Liu M., Yue Y., He B., Wang J. (2025). Investigation of the Differences in Amyloid-Like Fibrils Derived from Wheat Gluten with Varying Structures under Typical Food Processing Conditions. J. Agric. Food Chem..

[12] Garavand F., Khodaei D., Mahmud N., Islam J., Khan I., Jafarzadeh S., Tahergorabi R., Cacciotti I. (2024). Recent progress in using zein nanoparticles-loaded nanocomposites for food packaging applications. Crit. Rev. Food Sci. Nutr..

[13] He J., Tang H., Liao R., Lin H., Zhang W. (2025). Gemini surfactant stabilized zein nanoparticles: Preparation, characterization, interaction mechanism, and antibacterial activity. Int. J. Biol. Macromol..

[14] de Almeida Campos L.A., Neto A.F.S., Noronha M.C.S., de Lima M.F., Cavalcanti I.M.F., Santos-Magalhães N.S. (2023). Zein nanoparticles for drug delivery: Preparation methods and biological applications. Int. J. Pharm..

[15] Hsu W.-H., Ku C.-L., Lai Y.-R., Wang S.S.-S., Chou S.-H., Lin T.-H. (2023). Developing targeted drug delivery carriers for breast cancer using glutathione-sensitive doxorubicin-coupled glycated bovine serum albumin nanoparticles. Int. J. Biol. Macromol..

[16] Luque-Alcaraz A.G., Velazquez-Antillón M., Hernández-Téllez C.N., Graciano-Verdugo A.Z., García-Flores N., Iriqui-Razcón J.L., Silvas-García M.I., Zazueta-Raynaud A., Moreno-Vásquez M.J., Hernández-Abril P.A. (2022). Antioxidant effect of nanoparticles composed of zein and orange (*Citrus sinensis*) extract obtained by ultrasound-assisted extraction. Materials.

[17] Lai Y.-R., Huang C.-F., How S.-C., Lin T.-H., Wang S.S.-S. (2024). Using titanium dioxide nanoparticle-deposited whey protein isolate amyloid fibrils to photocatalyze the degradation of methylene blue. J. Taiwan Inst. Chem. Eng..

[18] Lai Y.-R., Wang T.-H., How S.-C., Lin K.-S., Chou W.-L., Wang S.S.-S. (2022). Using sugar-derived nanoparticles to mitigate amyloid fibril formation of lysozyme. J. Taiwan Inst. Chem. Eng..

[19] Danaei M., Dehghankhold M., Ataei S., Hasanzadeh Davarani F., Javanmard R., Dokhani A., Khorasani S., Mozafari M.R. (2018). Impact of particle size and polydispersity index on the clinical applications of lipidic nanocarrier systems. Pharmaceutics.

[20] Yeap S.P., Lim J., Ngang H.P., Ooi B.S., Ahmad A.L. (2018). Role of particle–particle interaction towards effective interpretation of Z-average and particle size distributions from dynamic light scattering (DLS) analysis. J. Nanosci. Nanotechnol..

[21] Wieser H., Koehler P., Scherf K.A. (2023). Chemistry of wheat gluten proteins: Qualitative composition. Cereal Chem..

[22] Liang Y., Song J., Wang J., Liu H., Wu X., He B., Zhang X., Wang J. (2023). Investigating the effects of NaCl on the formation of AFs from gluten in cooked wheat noodles. Int. J. Mol. Sci..

[23] Lai Y.-R., Wang S.S.-S., Hsu T.-L., Chou S.-H., How S.-C., Lin T.-H. (2023). Application of amyloid-based hybrid membranes in drug delivery. Polymers.

[24] Nazmi N., Isa M., Sarbon N. (2017). Preparation and characterization of chicken skin gelatin/CMC composite film as compared to bovine gelatin film. Food Biosci..

[25] Buhus G., Popa M., Desbrieres J. (2009). Hydrogels based on carboxymethylcellulose and gelatin for inclusion and release of chloramphenicol. J. Bioact. Compat. Polym..

[26] Azevedo V.M., Borges S.V., Marconcini J.M., Yoshida M.I., Neto A.R.S., Pereira T.C., Pereira C.F.G. (2017). Effect of replacement of corn starch by whey protein isolate in biodegradable film blends obtained by extrusion. Carbohydr. Polym..

[27] Omrani-Fard H., Abbaspour-Fard M.H., Khojastehpour M., Dashti A. (2020). Gelatin/whey protein-potato flour bioplastics: Fabrication and evaluation. J. Polym. Environ..

[28] Lai Y.-R., Hou X.-X., How S.-C., Lin T.-H., Wang S.S.-S. (2024). Development of two-dimensional amyloid fibril/carboxymethyl cellulose hybrid membranes for effective adsorption of hexavalent chromium. J. Environ. Chem. Eng..

[29] Chen Y., Tao X., He R., Ju X., Wang Z. (2025). High-flux and durable Janus membrane for oil-in-water emulsions separation via asymmetric acylated zein nanoparticle assembly. Sep. Purif. Technol..

[30] Teng D., Zhao T., Xu Y., Zhang X., Zeng Y. (2021). The zein-based fiber membrane with switchable superwettability for on-demand oil/water separation. Sep. Purif. Technol..

[31] Yang Y., Chen X., Li Y., Yin Z., Bao M. (2021). Construction of a superhydrophobic sodium alginate aerogel for efficient oil absorption and emulsion separation. Langmuir.

[32] Li Z., Wu J., Yue X., Qiu F., Yang D., Zhang T. (2020). Study on the application of waste bricks in emulsified oil-water separation. J. Clean. Prod..

[33] Sutrisna P.D., Kurnia K.A., Siagian U.W., Ismadji S., Wenten I.G. (2022). Membrane fouling and fouling mitigation in oil–water separation: A review. J. Environ. Chem. Eng..

[34] Agarwal S., von Arnim V., Stegmaier T., Planck H., Agarwal A. (2013). Role of surface wettability and roughness in emulsion separation. Sep. Purif. Technol..

[35] Zhu Y., Wang D., Jiang L., Jin J. (2014). Recent progress in developing advanced membranes for emulsified oil/water separation. NPG Asia Mater..

[36] Fang S., Wang Y., Zhu L., Zhang Y., Yu L.L. (2025). Effect of zein nanoparticles addition on anthocyanin and lutein dual-loaded nanocomposite hydrogels: Structure, physico-chemical and delivery properties. Int. J. Biol. Macromol..

[37] Hernández-Abril P.A., Luque-Alcaraz A.G., Iriqui-Razcón J.L., Higuera-Valenzuela H.J., Hernández-Tellez C.N. (2024). Understanding the Relationship Between Zein Solution Concentration and Nanoparticle Physicochemical Characteristics for Biomedical Use.

[38] Pan K., Zhong Q. (2016). Low energy, organic solvent-free co-assembly of zein and caseinate to prepare stable dispersions. Food Hydrocoll..

[39] Liu Q., Cheng J., Sun X., Guo M. (2021). Preparation, characterization, and antioxidant activity of zein nanoparticles stabilized by whey protein nanofibrils. Int. J. Biol. Macromol..

[40] Meewan J., Somani S., Almowalad J., Laskar P., Mullin M., MacKenzie G., Khadke S., Perrie Y., Dufès C. (2022). Preparation of zein-based nanoparticles: Nanoprecipitation versus microfluidic-assisted manufacture, effects of PEGylation on nanoparticle characteristics and cellular uptake by melanoma cells. Int. J. Nanomed..

[41] Luque-Alcaraz A.G., Maldonado-Arriola J.A., Hernández-Abril P.A., Álvarez-Ramos M.E., Hernández-Téllez C.N. (2025). Zein Nanoparticles Loaded with Vitis vinifera L. Grape Pomace Extract: Synthesis and Characterization. Nanomaterials.

[42] Jiang Y.-H., Zhang Y.-Q., Gao C., An Q.-D., Xiao Z.-Y., Zhai S.-R. (2022). Superhydrophobic aerogel membrane with integrated functions of biopolymers for efficient oil/water separation. Sep. Purif. Technol..

[43] Lei S., Zeng M., Huang D., Wang L., Zhang L., Xi B., Ma W., Chen G., Cheng Z. (2019). Synergistic high-flux oil–saltwater separation and membrane desalination with carbon quantum dots functionalized membrane. ACS Sustain. Chem. Eng..

[44] Zhang Y., Zhang Y., Cao Q., Wang C., Yang C., Li Y., Zhou J. (2020). Novel porous oil-water separation material with super-hydrophobicity and super-oleophilicity prepared from beeswax, lignin, and cotton. Sci. Total Environ..

[45] Xue J., Zhu L., Zhu X., Li H., Ma C., Yu S., Sun D., Xia F., Xue Q. (2021). Tetradecylamine-MXene functionalized melamine sponge for effective oil/water separation and selective oil adsorption. Sep. Purif. Technol..

[46] Li Y., Zhang Z., Ge B., Men X., Xue Q. (2017). A versatile and efficient approach to separate both surfactant-stabilized water-in-oil and oil-in-water emulsions. Sep. Purif. Technol..

[47] Guo F., Wen Q., Guo Z. (2017). Low cost and non-fluoride flowerlike superhydrophobic particles fabricated for both emulsions separation and dyes adsorption. J. Colloid Interface Sci..

[48] Xiang Q., Liu Y., Wang B., Huang C., Wang L., He J., Tian D., Shen F., Zhang Y. (2025). A universal strategy for efficient separation from single emulsion separation to oil-in-water and water-in-oil mixed emulsions. Sep. Purif. Technol..

[49] Zhang Q., Li K., Li J., Li Y. (2024). Fabrication of hierarchically porous superhydrophobic polystyrene foam for self-cleaning, oil absorbent, highly efficient oil–water separation. Chem. Eng. J..

[50] Cao W., Zhang M., Ma W., Huang C. (2023). Multifunctional electrospun nanofibrous membrane: An effective method for water purification. Sep. Purif. Technol..

[51] Langer R. (1980). Invited review polymeric delivery systems for controlled drug release. Chem. Eng. Commun..

[52] Lai Y.-R., Ho T.-L., Yang Y.-H., Lin T.-H., Wang S.S.-S. (2025). Development of sustainable bioplastic films for food packaging using zein protein-derived amyloid fibrils: Characterization and functional properties. Int. J. Biol. Macromol..

[53] Ejeta D.D., Wang C.-F., Kuo S.-W., Chen J.-K., Tsai H.-C., Hung W.-S., Hu C.-C., Lai J.-Y. (2020). Preparation of superhydrophobic and superoleophilic cotton-based material for extremely high flux water-in-oil emulsion separation. Chem. Eng. J..

[54] Upadhyaya L., Qian X., Wickramasinghe S.R. (2018). Chemical modification of membrane surface—Overview. Curr. Opin. Chem. Eng..

