# Supplementary material for: Fabrication of Zein Nanoparticle-Functionalized Wheat Gluten Amyloid Fibril/Methyl Cellulose Hybrid Membranes with Efficient Performance for Water-in-Oil Emulsion Separation
Source: Polymers (Basel). 2025 Sep 4;17(17):2409. doi: 10.3390/polym17172409 (PMC12431481; doi:10.3390/polym17172409)
Supplement: Supplementary file 1 [file polymers-17-02409-s001.zip › polymers-3820450-supplementary.pdf]

Supporting Information

Fabrication of zein nanoparticle-functionalized wheat gluten amyloid fibril/methyl cellulose hybrid membranes with efficient performance for water-in-oil emulsion separation

You-Ren Lai [1,†], Jun-Ying Lin [1,†], Jou-Ting Hsu [1], Ta-Hsien Lin [2], Su-Chun How [3,*] and Steven S.-S. Wang [1,*]

[1] Department of Chemical Engineering, National Taiwan University, Taipei 106319, Taiwan; ray110135@gmail.com (Y.-R.L.); kuroha56780@gmail.com (J.-Y.L.); 710416wlsh.tyc.edu.tw@gmail.com (J.-T.H.)

[2] Institute of Biochemistry and Molecular Biology, National Yang Ming Chiao Tung University, Taipei 11221, Taiwan; thlin@vghtpe.gov.tw

[3] Department of Chemical Engineering and Biotechnology, Tatung University, Taipei 10452, Taiwan

* Correspondence: schow@gm.ttu.edu.tw (S.-C.H.); sswang@ntu.edu.tw (S.S.-S.W.); Tel.: +886-2-2182-2928 (S.-C.H.); +886-2-3366-5870 (S.S.-S.W.)

† These authors contributed equally to this work.

Table S1. Estimate of average hydrodynamic diameters for various emulsions before and after filtering through MC, WGAF/MC-1, WGAF/MC-2, and ZNP-WGAF/MC membranes.

Membranes \ Emulsions	Average hydrodynamic diameter for emulsified W/O droplets				
	**GOL**	**DSO**	**SBO**	**GRO**	**SFO**
**Before filtration**	14735	4510	19640	1492	25750
**MC**	1170	947.6	1467	868.4	1373
**WGAF/MC-1**	201.8	1.568	5.963	312.5	571.6
**WGAF/MC-2**	37.89	1.211	4.134	5.101	3.749
**ZNP-WGAF/MC**	21.77	0.946	0.654	6.388	0.673

Table S2. A summary of the synthesis methods and particle sizes of various zein nanoparticles.

Samples	***Synthesis methods***	***Particle size***	***Ref.***
Lutein-loaded zein nanoparticles	Antisolvent precipitation	D_h = 78.13 ± 0.67 nm	[1]
Zein nanoparticles	Antisolvent method	D_h = ~180 nm	[2]
Zein–NaCas	pH-cycle method	D_h = 96.9 ± 6.6 nm	[3]
WPNZ-NPs	pH-driven method	D_h = ~410 nm	[4]
mPEG5K-zein	Microfluidic approach	D_h = 133.3 ± 1.7 nm	[5]
ZNP-GPE	Antisolvent precipitation	D_h = 138.2 ± 1.49 nm	[6]
NpZOE	Antisolvent method	D_h = 199.96 ± 2.87 nm	[7]
Zein nanoparticles	Antisolvent method	D_h = 37.43 ± 6.37 nm	Present work

Abbreviation: NaCas–sodium caseinate; WPNZ-NPs–whey protein nanofibrils stabilized zein nanoparticles; mPEG5K–methoxy PEG succinimidyl carboxymethyl ester (MW = 5 kDa); GPE–grape pomace extract; NpZOE–zein nanoparticles with orange extract.

Table S3. Comparison of the performances of emulsion separation systems using various materials.

Materials	Synthesis methods	Oil-water mixture	Separation efficiency (%)	Size of emulsion after filtration	Ref.
T-SA/lignin^x/rGO-MTMS aerogel membrane	Chelation & CVD & lyophilization	Chloroform-water	~96.7	—	[8]
C_{18}-CQDs membrane	Thermal decomposition method	(1) hexane-water<br>(2) dodecane-water	(1) >99 %<br>(2) >99 %	—	[9]
Biomass-based porous materials (cotton, beeswax, and lignin)	Surface coating	Trichloromethane-water	~93.78 %	—	[10]
TDA-MXene@MS	Surface functionalization & vacuum drying	(1) water-in-toluene<br>(2) water-in-dichloromethane	(1) ~96.88 %<br>(2) ~97.10 %	—	[11]
Nanoporous DVB/SiO_2 hybrid material	Solvothermal method	(1) water-in-toluene<br>(2) water-in-chloroform<br>(3) water-in-hexane	—	(1) ~1 nm<br>(2) ~40–100 nm<br>(3) ~100–1000 nm	[12]
Green fluoride-free superhydrophobic hierarchical flowerlike iron-containing MnO_2 particles	One-pot approach & stearic acid modification	(1) diesel-in-water<br>(2) isooctane-in-water	Change in turbidity of samples before and after filtration		[13]
Treated bamboo powder loaded filtration devices	Assembled with polyvinyl chloride molds and quartz tubes	(1) water-in-diesel<br>(2) water-in-gasoline<br>(3) water-in-kerosene<br>(4) water-in-hexane	(1) >99 %<br>(2) >99 %<br>(3) >99 %<br>(4) >99 %	(1) ~0.5–2 nm<br>(2) ~1–10 nm<br>(3) ~4–40 nm<br>(4) ~4–20 nm	[14]

		(5) water-in-petroleum ether	(5) >99 %	(5) ~1–20 nm	
WPIAF-CMC aerogel	Salting-out method & lyophilization	(1) water-in-gear oil<br>(2) water-in-soybean oil<br>(3) water-in-gasoline<br>(4) water-in-diesel oil	(1) ~96.04 %<br>(2) ~94.32 %<br>(3) ~95.03 %<br>(4) ~94.02 %	(1) ~0.5–1 nm<br>(2) ~0.5–4 nm<br>(3) ~1–4 nm<br>(4) ~0.4–1 nm	[15]
WPIAF-CS aerogel	Salting-out method & lyophilization	(1) water-in-gear oil<br>(2) water-in-soybean oil<br>(3) water-in-gasoline<br>(4) water-in-diesel oil	(1) ~97.03 %<br>(2) ~97.46 %<br>(3) ~95.79 %<br>(4) ~91.52 %	(1) ~1–3 nm<br>(2) ~0.5–10 nm<br>(3) ~8–20 nm<br>(4) ~1–3 nm	[15]
WGAF/MC membrane	Chemical crosslinking & casting method	(1) water-in-gear oil<br>(2) water-in-soybean oil<br>(3) water-in-gasoline<br>(4) water-in-diesel oil<br>(5) water-in-sunflower oil	(1) ~95.96 %<br>(2) ~99.22 %<br>(3) ~85.12 %<br>(4) ~96.83 %<br>(5) ~92.74 %	(1) ~5.1 nm<br>(2) ~4.1 nm<br>(3) ~37.9 nm<br>(4) ~1.2 nm<br>(5) ~3.7 nm	This work
ZNP-WGAF/MC membrane	Chemical crosslinking & casting method	(1) water-in-gear oil<br>(2) water-in-soybean oil<br>(3) water-in-gasoline<br>(4) water-in-diesel oil<br>(5) water-in-sunflower oil	(1) ~91.26 %<br>(2) ~99.44 %<br>(3) ~87.17 %<br>(4) ~97.59 %<br>(5) ~98.52 %	(1) ~6.4 nm<br>(2) ~0.6 nm<br>(3) ~21.7 nm<br>(4) ~0.9 nm<br>(5) ~0.6 nm	This work

Abbreviations: WGAF – whey gluten amyloid fibril; MC –methyl cellulose; ZNP – zein nanoparticle; WPIAF – whey protein isolate amyloid fibril; CMC –carboxymethyl cellulose; CS – chitosan; TDA –tetradecylamine; MS – melamine sponge; C_{18}-CQDs – octadecylamine-functionalized carbon quantum dots; GO – graphene oxide; SA – sodium alginate; MTMS – methyltrimethoxysilane; DVB – divinylbenzene.

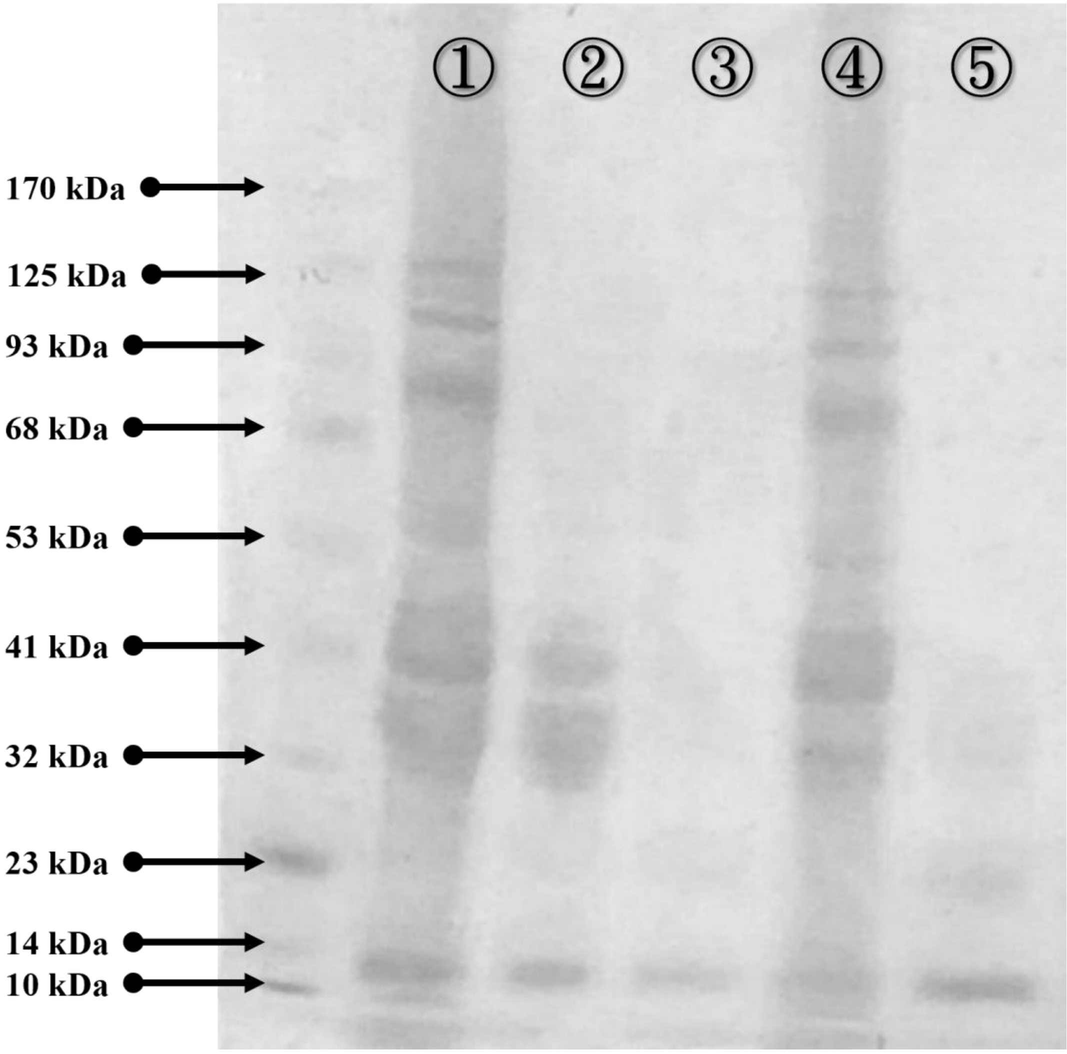

Figure S1. (A) SDS-PAGE analyses of WG (Lane 1: unpurified WG (6 mg/mL); Lane 2: supernatant obtained after purification with a 70% ethanol solution (6 mg/mL); Lane 3: supernatant obtained after purification with a 0.5% SDS-PB buffer (6 mg/mL); Lane 4: purified WG (6 mg/mL); Lane 5: supernatant obtained after purification with a 0.5% SDS-PB buffer (24 mg/mL)).

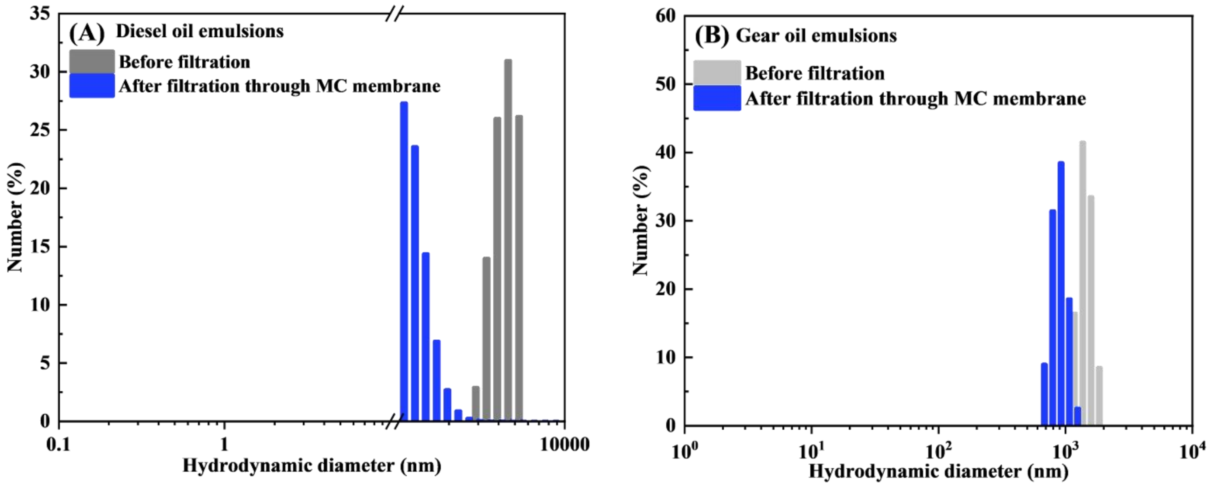

Figure S2. Hydrodynamic size distributions of the W/O emulsions (A: diesel oil and B: gear oil) before and after filtration through MC membrane.

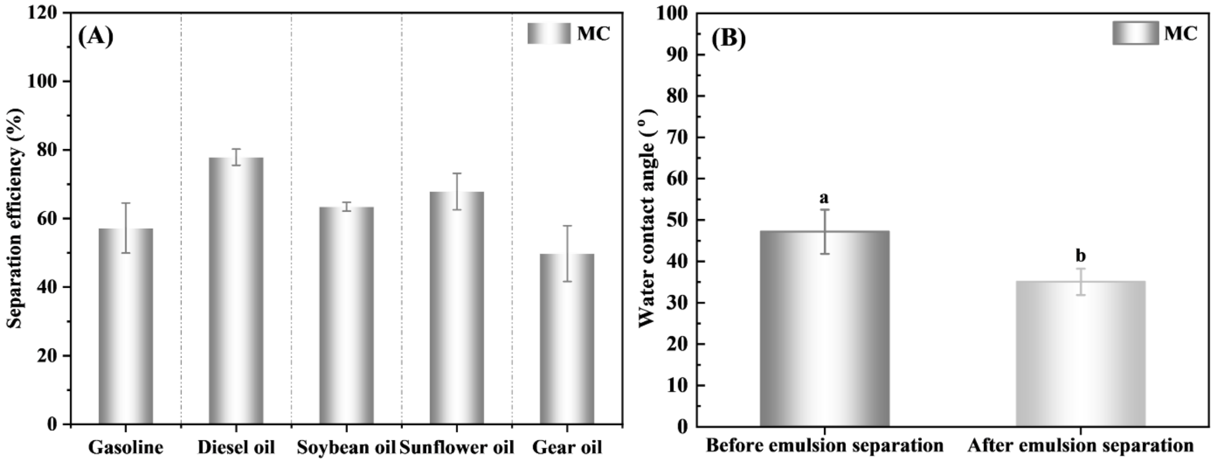

Figure S3. (A) Separation performances of the MC membrane for various emulsified W/O droplets. (B) Water contact angles of the MC membrane before and after filtering emulsified W/DSO mixtures. (Different lowercase letters above bars show significant differences ($p<0.05$))

Reference

1. Fang, S.; Wang, Y.; Zhu, L.; Zhang, Y.; Yu, L. L., Effect of zein nanoparticles addition on anthocyanin and lutein dual-loaded nanocomposite hydrogels: Structure, physico-chemical and delivery properties. *International Journal of Biological Macromolecules* **2025,** *309*, 142967.

2. Hernández-Abril, P. A.; Luque-Alcaraz, A. G.; Iriqui-Razcón, J. L.; Higuera-Valenzuela, H. J.; Hernández-Tellez, C. N., Understanding the Relationship Between Zein Solution Concentration and Nanoparticle Physicochemical Characteristics for Biomedical Use. Oxford University Press US: 2024.

3. Pan, K.; Zhong, Q., Low energy, organic solvent-free co-assembly of zein and caseinate to prepare stable dispersions. *Food Hydrocolloids* **2016,** *52*, 600-606.

4. Liu, Q.; Cheng, J.; Sun, X.; Guo, M., Preparation, characterization, and antioxidant activity of zein nanoparticles stabilized by whey protein nanofibrils. *International Journal of Biological Macromolecules* **2021,** *167*, 862-870.

5. Meewan, J.; Somani, S.; Almowalad, J.; Laskar, P.; Mullin, M.; MacKenzie, G.; Khadke, S.; Perrie, Y.; Dufès, C., Preparation of zein-based nanoparticles: Nanoprecipitation versus microfluidic-assisted manufacture, effects of PEGylation on nanoparticle characteristics and cellular uptake by melanoma cells. *International journal of nanomedicine* **2022**, 2809-2822.

6. Luque-Alcaraz, A. G.; Maldonado-Arriola, J. A.; Hernández-Abril, P. A.; Álvarez-Ramos, M. E.; Hernández-Téllez, C. N., Zein Nanoparticles Loaded with Vitis vinifera L. Grape Pomace Extract: Synthesis and Characterization. *Nanomaterials* **2025,** *15* (7), 539.

7. Luque-Alcaraz, A. G.; Velazquez-Antillón, M.; Hernández-Téllez, C. N.; Graciano-Verdugo, A. Z.; García-Flores, N.; Iriqui-Razcón, J. L.; Silvas-García, M. I.; Zazueta-Raynaud, A.; Moreno-Vásquez, M. J.; Hernández-Abril, P. A., Antioxidant effect of nanoparticles composed of zein and orange (citrus sinensis) extract obtained by ultrasound-assisted extraction. *Materials* **2022,** *15* (14), 4838.

8. Jiang, Y.-H.; Zhang, Y.-Q.; Gao, C.; An, Q.-D.; Xiao, Z.-Y.; Zhai, S.-R., Superhydrophobic aerogel membrane with integrated functions of biopolymers for efficient oil/water separation. *Separation and purification technology* **2022,** *282*, 120138.

9. Lei, S.; Zeng, M.; Huang, D.; Wang, L.; Zhang, L.; Xi, B.; Ma, W.; Chen, G.; Cheng, Z., Synergistic high-flux oil–saltwater separation and membrane desalination with carbon quantum dots functionalized membrane. *ACS Sustainable Chemistry & Engineering* **2019,** *7* (16), 13708-13716.

10. Zhang, Y.; Zhang, Y.; Cao, Q.; Wang, C.; Yang, C.; Li, Y.; Zhou, J., Novel porous oil-water separation material with super-hydrophobicity and super-oleophilicity prepared from beeswax, lignin, and cotton. *Science of the total environment* **2020,** *706*, 135807.

11. Xue, J.; Zhu, L.; Zhu, X.; Li, H.; Ma, C.; Yu, S.; Sun, D.; Xia, F.; Xue, Q., Tetradecylamine-MXene functionalized melamine sponge for effective oil/water separation and selective oil adsorption. *Separation and Purification Technology* **2021,** *259*, 118106.

12. Li, Y.; Zhang, Z.; Ge, B.; Men, X.; Xue, Q., A versatile and efficient approach to separate both surfactant-stabilized water-in-oil and oil-in-water emulsions. *Separation and Purification Technology* **2017,** *176*, 1-7.

13. Guo, F.; Wen, Q.; Guo, Z., Low cost and non-fluoride flowerlike superhydrophobic particles fabricated for both emulsions separation and dyes adsorption. *Journal of Colloid and Interface Science* **2017,** *507*, 421-428.

14. Xiang, Q.; Liu, Y.; Wang, B.; Huang, C.; Wang, L.; He, J.; Tian, D.; Shen, F.; Zhang, Y., A universal strategy for efficient separation from single emulsion separation to oil-in-water and water-in-oil mixed emulsions. *Separation and Purification Technology* **2025,** *354*, 129517.

15. Tu, J.-L.; Lai, Y.-R.; Lin, C.-Y.; Wang, S. S.-S.; Lin, T.-H., Applications of three-dimensional whey protein amyloid fibril-based hybrid aerogels in oil/water separation and emulsion separation. *International Journal of Biological Macromolecules* **2024,** *283*, 137680.
